# Detection and genetic characterization of a wide range of infectious agents in *Ixodes pavlovskyi* ticks in Western Siberia, Russia

**DOI:** 10.1186/s13071-017-2186-5

**Published:** 2017-05-25

**Authors:** Vera Rar, Natalia Livanova, Sergey Tkachev, Galina Kaverina, Artem Tikunov, Yuliya Sabitova, Yana Igolkina, Victor Panov, Stanislav Livanov, Nataliya Fomenko, Igor Babkin, Nina Tikunova

**Affiliations:** 1 Institute of Chemical Biology and Fundamental Medicine, SB RAS, Novosibirsk, Russian Federation; 2 Institute of Systematics and Ecology of Animals SB RAS, Novosibirsk, Russian Federation

**Keywords:** *Ixodes pavlovskyi*, *Ixodes persulcatus*, Tick-borne encephalitis virus, Kemerovo virus, *Borrelia burgdorferi* (*sensu lato*), *Borrelia miyamotoi*, *Rickettsia* spp, Anaplasmataceae, *Babesia microti*, Western Siberia

## Abstract

**Background:**

The *Ixodes pavlovskyi* tick species, a member of the *I. persulcatus/I. ricinus* group, was discovered in the middle of the 20^th^ century in the Russian Far East. Limited data have been reported on the detection of infectious agents in this tick species. The aim of this study was to investigate the prevalence and genetic variability of a wide range of infectious agents in *I. pavlovskyi* ticks collected in their traditional and recently invaded habitats, the Altai Mountains and Novosibirsk Province, respectively, which are both located within the Western Siberian part of the *I. pavlovskyi* distribution area.

**Results:**

This study reports the novel discovery of *Borrelia bavariensis*, *Rickettsia helvetica*, *R. heilongjiangensis*, *R. raoultii*, “*Candidatus* Rickettsia tarasevichiae”, *Anaplasma phagocytophilum*, *Ehrlichia muris*, “*Candidatus* Neoehrlichia mikurensis” and *Babesia microti* in *I. pavlovskyi* ticks. In addition, we confirmed the previous identification of *B. afzelii*, *B. garinii* and *B. miyamotoi*, as well as tick-borne encephalitis and Kemerovo viruses in this tick species. The prevalence and some genetic characteristics of all of the tested agents were compared with those found in *I. persulcatus* ticks that were collected at the same time in the same locations, where these tick species occur in sympatry. It was shown that the prevalence and genotypes of many of the identified pathogens did not significantly differ between *I. pavlovskyi* and *I. persulcatus* ticks. However, *I. pavlovskyi* ticks were significantly more often infected by *B. garinii* and less often by *B. bavariensis*, *B. afzelii*, “*Ca*. R. tarasevichiae”, and *E. muris* than *I. persulcatus* ticks in both studied regions. Moreover, new genetic variants of *B. burgdorferi* (*sensu lato*) and *Rickettsia* spp. as well as tick-borne encephalitis and Kemerovo viruses were found in both *I. pavlovskyi* and *I. persulcatus* ticks.

**Conclusion:**

Almost all pathogens that were previously detected in *I. persulcatus* ticks were identified in *I. pavlovskyi* ticks; however, the distribution of species belonging to the *B. burgdorferi* (*sensu lato*) complex, the genus *Rickettsia*, and the family *Anaplasmataceae* was different between the two tick species. Several new genetic variants of viral and bacterial agents were identified in *I. pavlovskyi* and *I. persulcatus* ticks.

## Background

In the Northern Hemisphere, at least five tick species of the genus *Ixodes* (Ixodidae) can transmit a great variety of infectious agents to humans: *I. ricinus*, *I. persulcatus*, *I. scapularis*, *I. ovatus*, *I. pacificus* and *I. hexagonus*. The most important pathogens vectored by these *Ixodes* ticks are a number of bacteria of the *Borrelia burgdorferi* (*sensu lato*) (*s.l*.) complex and tick-borne encephalitis virus (TBEV) from the family *Flaviviridae* [1–11]. In addition, the causative agents of rickettsioses, relapsing fever borreliosis, ehrlichiosis, anaplasmosis, neoehrlichiosis, babesiosis, tularemia and bartonellosis can be detected in these tick species [9, 10, 12–22]. Moreover, a number of pathogens of veterinary importance can also be vectored by these *Ixodes* ticks [23, 24]. A large number of studies of the ecology, geographical distribution, and genetic variability of *I. ricinus*, *I. scapularis* and *I. pacificus* ticks and the molecular epidemiology of pathogens transmitted by them have been published [4, 7, 9, 10, 12, 25–28]. The ability of *I. persulcatus* ticks to transmit the Far Eastern subtype of TBEV, which causes a severe neurological disease, and the wide distribution area of this tick species have led to the sustained attention of investigators from Russia on this tick species. This has resulted in the accumulation of data on the biology, occurrence and medical importance of *I. persulcatus* ticks, although some of this information is available only in the Russian scientific literature [1, 3, 29–38]. In Russia, TBEV, Kemerovo virus (KEMV), *B. afzelii*, *B. bavariensis*, *B. garinii*, *B. valaisiana*, *B. miyamotoi*, *Rickettsia heilongjiangensis*, *R. helvetica*, *R. raoultii*, *R. sibirica*, “*Candidatus* Rickettsia tarasevichiae”, *Anaplasma phagocytophilum*, *Ehrlichia muris*, “*Candidatus* Neoehrlichia mikurensis*”*, *Babesia microti*, *Bab. venatorum* and *Bartonella* spp*.* have all been found in *I. persulcatus* ticks [3, 5, 14, 15, 39–51].

In the middle of the 20^th^ century, a new species of *Ixodes* ticks, *I. pavlovskyi*, was discovered in the Russian Far East [52]. This tick species, belonging to the *I. persulcatus*/*I. ricinus* group, has a discontinuous distribution area, including the Far Eastern (southern areas of the Russian Far East Manchuria in China and northern regions in Japan) and Western Siberian (Altai and Kuznetsk Alatau Mountains and Salair Ridge) regions [1, 53]. In the last century, single *I. pavlovskyi* ticks have been recorded in more northern sites located in the Western Siberian Plain, but these findings have been rare [29]. *Ixodes pavlovskyi* ticks are morphologically and genetically similar to *I. persulcatus*, occur in sympatry, and have a comparable ecology [54–58]. Their activity seasons overlap, and larvae and nymphs of both tick species usually feed on the same hosts [1]. However, *I. persulcatus* adults feed on large and medium-sized wild mammals and livestock, while *I. pavlovskyi* adults feed on birds that collect food from the ground and have been found to feed on the European hedgehog (*Erinaceus europaeus*), the mountain hare (*Lepus timidus*), and the red squirrel (*Sciurus vulgaris*) [59, 60]. Notably, natural hybridization between *I. pavlovskyi* and *I. persulcatus* ticks in their sympatric populations in Western Siberia has been described [61]. From the beginning of this century, an increased abundance of *I. pavlovskyi* ticks has been recorded more northward in Western Siberia in parks and suburban areas of Novosibirsk and Tomsk, large Siberian cities situated in the Western Siberian Plain. In these suburban areas, *I. pavlovskyi* ticks have become predominant in a number of locations, reaching 82–94% of tick samplings [35, 58, 62, 63]. *Ixodes pavlovskyi* ticks frequently attack people [1, 64, 65]; however, the role of the tick species in the epidemiology of tick-borne diseases has not been studied. The natural locations inhabited by sympatric populations of *I. pavlovskyi* and *I. persulcatus* are poorly characterized. The cause of the recent expansion of *I. pavlovskyi* ticks is unknown. Limited data have been reported on the detection of tick-borne pathogens in these ticks, including TBEV, KEMV, *B. afzelii*, *B. garinii* and *B. miyamotoi* [36, 43, 48, 51, 66, 67]. In addition, DNA of *A. phagocytophilum* and “*Ca.* N. mikurensis” were identified in *I. pavlovskyi* ticks when a bacterial community associated with this tick species was studied by metagenomics 16S profiling [68].

In this study, a wide range of infectious agents was investigated in *I. pavlovskyi* ticks collected in their previously known habitat in the Northern part of the Altai Mountains, as well as in their recently recorded habitat near Novosibirsk, Western Siberia, Russia. In addition, the prevalence and genetic divergence of detected agents were compared with those found in well-known *I. persulcatus* ticks that were simultaneously caught in the same locations.

## Methods

### Field study

**Fig. 1:**
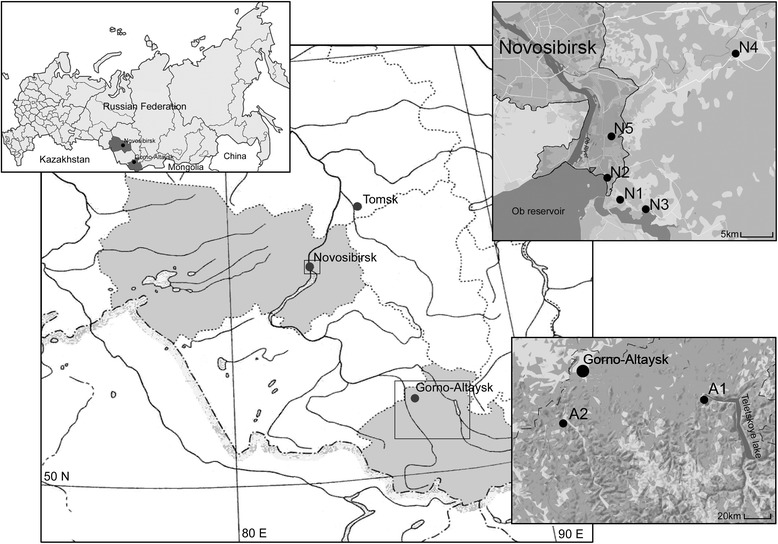
Sites of tick collections in Western Siberia. *Legend*: A1-A2, sites located in the Republic of Altai; N1-N5, sites located in Novosibirsk Province

Questing *Ixodes* spp. ticks were collected by flagging along linear transects in May-June of 2010–2015 in two locations in Western Siberia, Russia: in the northern part of the Altai Mountains, Republic of Altai (two sites) and in parks and suburbs of the city of Novosibirsk, Novosibirsk Province (five sites) (Fig. 1). The description of the sampling sites is given in Table 1.

**Table 1:** Collection of *I. pavlovskyi* and *I. persulcatus* ticks in different sites

Region (Year)	Site	Coordi-nates	Biotope description	No. of collected ticks	Tick species	No. of ticks identified morphologically	No. of ticks identified both morphologically and genetically/% of collected ticks
Alt (2012, 2014, 2015)	A1	51°47'N, 87°18'E	Mountain slopes near Artybash village, Turochaksky District; overgrown old pathways in mixed forests of *Abies sibírica*, *Betula pendula* and *Pinus sibirica*	55	*I. pavl*	20	11/20.0
					*I. pers*	35	33/60.0
	A2	51°36'N, 85°48'E	Mountain slopes near Altai department of Central Siberian Botanic Garden, Shebalinsky District; overgrown old pathways in mixed forest of *Pinus sylvestris* and *Betula pendula*	998	*I. pavl*	142	113/11.3
					*I. pers*	823 (189)^a^	152/nd
	Total A1-A2			1053	*I. pavl*	162	124/11.8
					*I. pers*	858 (224)^a^	185/nd
Nov (2010, 2014, 2015)	N1	54°48'N, 83°07'E	Central Siberian Botanic Garden, Sovetsky District of Novosibirsk; overgrown pathways in mixed forest of *Betula pendula* and *Pinus sylvestris*	448	*I. pavl*	381	316/70.5
					*I. pers*	32	22/4.9
	N2	54°50'N, 83°05'E	Floodplain of Zyryanka rivulet, Sovetsky District of Novosibirsk (AT); overgrown pathways in mixed forest of *Betula pendula* and *Pinus sylvestris*	27	*I. pavl*	27	22/81.5
					*I. pers*	0	0
	N3	54°46'N, 83°09'E	Mouth of Shadrikha River (SR) Novosibirsky District; mixed forest of *Pinus sylvestris* and *Betula pendula*	433	*I. pavl*	157	79/18.2
					*I. pers*	236	110/25.4
	N4	55°00'N, 83°24'E	Ravine near Plotnikovo village (PV) Novosibirsky District; with *Prunus padus*, *Populus tremula* and *Amelanchier* spp.	64	*I. pavl*	39	22/34.4
					*I. pers*	25	14/21.9
	N5	54°53'N, 83°08'E	Surroundings of Nizhnaya Yeltsovka district of Novosibirsk; young birch stands on reforesting crop fields and small old birch stands with *Populus tremula* and *Pinus sylvestris*	24	*I. pavl*	18	14/58.3
					*I. pers*	5	3/12.5
	Total N1-N5			996	*I. pavl*	622	453/45.5
					*I. pers*	298	149/15.0
All sites				2049	*I. pavl*	784	577/20.7
					*I. pers*	1156 (522)^a^	334/nd

*Abbreviations*: *Alt* Republic of Altai, *Nov* Novosibirsk Province, *nd* not detected, *I. pavl Ixodes pavlovskyi*, *I. pers I. persulcatus*

^a^Numbers of *I. persulcatus* ticks subjected for genetic analysis are given in parentheses

The species, sex, and stage of collected ticks were determined using a binocular microscope, according to morphological keys [1]. Differentiation of *I. pavlovskyi* from *I. persulcatus* ticks was based on the following morphological criteria: conscutum color, scapular grooves profile, punctuations and form of the scutum, form of the auriculae, and form of the basis capituli. Adult ticks that could not be clearly identified as *I. pavlovskyi* or *I. persulcatus* as well as nymphs were excluded from this study.

### DNA extraction

To prevent cross-contamination, DNA extraction, amplification, and PCR product detection were carried out in separate rooms. Aerosol-free pipette tips were used at each stage. Ticks were individually washed with bi-distilled water, 70% ethanol and bi-distilled water once more. Afterwards, ticks were homogenized with a MagNA Lyser system (Roche Applied Science, Germany) and used for the isolation of total nucleic acids using a Proba NK kit (DNA-Technology, Moscow, Russia) according to the manufacturer’s protocol; nucleic acid samples were stored at -70 °C.

### Genetic characterization of ticks

Two genetic loci were used to confirm the species identities of *Ixodes* ticks: the mitochondrial cytochrome *c* oxidase subunit 1 (*cox*1) gene and the nuclear internal transcribed spacer (ITS2). For species determination based on the *cox*1 gene, species-specific PCR on the nucleic acid specimen of each tick was carried out with previously designed primers [69]: Ixodes-F and Ipers-R, specific to *I. persulcatus*, and Ixodes-F and Ipav-R, specific to *I. pavlovskyi* (Table 2). The lengths of the PCR fragments were 689–690 bp. In addition, sequencing of the nuclear genome fragment ITS2, amplified using the primers F-ITS2 and R1-ITS2 (Table 2), which have been described previously [58], was used for genetic characterization of each tick. The lengths of the PCR fragments were 632–636 bp.

**Table 2:** Primers used for PCR

Amplified locus	Primer sequences (5'-3')	Annealing temperature	Reference
*Ixodes* sp. ITS2	F-ITS2 (cacactgagcacttactctttg) R1-ITS2 (actggatggctccagtattc)	57 °C	[58]
*I. persulcatus cox*1 gene	Ixodes-F (acctgatatagctttccctcg) Ipers-R (ttgattcctgttggaacagc)	55 °C	[69]
*I. pavlovskyi cox*1 gene	Ixodes-F (acctgatatagctttccctcg) Ipav-R (taatccccgtggggacg)	55 °C	[69]
TBEV E-NS1 genes	E7 (ggcatagaaaggctgacagtg) E10 (gatacctctctccacacaaccag)	52 °C	[34]
	E9 (acagtgataggagaacacgcctggg) E8 (cagccaggaggaagctcatggac)	52 °C	[34]
KEMV segment 1	Kem1s_1 (attcaaattacgacacgcacatgac) Kem1s_2 (gtatcgtcgccgacgtacatctc)	56 °C	[70]
	Kem1s_3 (gctcatcgaagcgggatacgg) Kem1s_4 (gcgtagagttctctcccgacagatg)	56 °C	[70]
*Borrelia burgdorferi* (*s.l*.) 5S-23S rRNA intergenic spacer	NC1 (cctgttatcattccgaacacag) NC2 (tactccattcggtaatcttggg)	50 °C	[14]
	NC3 (tactgcgagttcgcgggag) NC4 (cctaggcattcaccatagac)	54 °C	[71], modified
*B. miyamotoi* *glpQ* gene	Q1 (caccattgatcatagctcacag) Q4 (ctgttggtgcttcattccagtc)	50 °C	[44]
	Q3 (gctagtgggtatcttccagaac) Q2 (cttgttgtttatgccagaagggt)	54 °C	[44]
*B. burgdorferi* (*s.l*.) *p83/100* gene	F7 (ttcaaagggatactgttagagag) F10 (aagaaggcttatctaatggtgatg)	50 °C	This study
	F5 (acctggtgatgtaagttctcc) F12 (ctaacctcattgttgttagactt)	54 °C	This study
*B. burgdorferi* (*s.l*.) *clpA* gene	clpAF1237 (aaagatagatttcttccagac) clpAR2218 (gaatttcatctattaaaagctttc)	55 → 48 °C	[72]
	clpAF1255 (gacaaagcttttgatattttag) clpAR2104 (caaaaaaaacatcaaattttctatctc)	50 °C	[72]
*Anaplasmataceae* 16S rRNA gene	Ehr1 (gaacgaacgctggcggcaagc) Ehr2 (agtaycgraccagatagccgc)	57 °C	[45]
	Ehr3 (tgcataggaatctacctagtag) Ehr4 (ctaggaattccgctatcctct)	60 °C	[45]
*A. phagocytophilum* 16S rRNA gene	HGE1 (cggattattctttatagcttgc) HGE2 (cttaccgaaccgcctacatg)	55 °C	[45]
*E. muris* 16S rRNA gene	Em1 (cgaacggatagctacccatagc) Em2 (cgctccaaagttaagctttggt)	55 °C	[45]
*Anaplasmataceae* *groESL* operon	HS1-f (cgycagtgggctggtaatgaa) HS6-r (ccwccwggtacwacaccttc)	55 °C	[73], modified
	HS3-f (atagtyatgaaggagagtgat) HSVR (tcaacagcagctctagtwg)	50 °C	[74]
*Rickettsia* spp. *gltA* gene	glt1 (gattgctttacttacgaccc) glt2 (tgcatttctttccattgtgc)	52 °C	[49]
	glt3 (tatagacggtgataaaggaatc) glt4 (cagaactaccgatttctttaagc)	53 °C	[49]
“*Ca.* R. tarasevichiae” *gltA* gene	RT1 (tactaaaaaagtcgctgttcattc) RT2 (tgttgcaaacatcatgcgtaag)	56 °C	[49]
SFGR *gltA*	RH1 (gtcagtctactatcacctatatag) RH3 (taaaatattcatctttaagagcga)	54 °C	[49] This study
*Babesia* spp. 18S rRNA gene	BS1 (gacggtagggtattggcct) BS2 (attcaccggatcactcgatc)	58 °C	[46]
	BS3 (taccggggcgacgacgggtg) BS5 (cgaggcagcaacgggtaacg) BS4 (agggacgtagtcggcacgag)	62 °C	[46]

### Detection and genotyping of KEMV and TBEV

cDNA was synthesized by reverse transcription performed using a RevertaL-100 kit containing random hexanucleotides (Amplisence, Moscow, Russia), with total nucleic acids isolated from ticks as the template. Primers specific to E-NS1 gene sequences of all TBEV subtypes were used for the primary (E7 and E10) and nested (E9 and E8) reactions (Table 2) [34]. For KEMV detection, primers specific to KEMV genome segment 1 sequences were designed for primary (Kem1s_1 and Kem1s_2) and nested (Kem1s_3 and Kem1s_4) reactions (Table 2) [70].

### *Borrelia* spp. nucleic acid detection

Detection of *Borrelia* DNA was carried out using multiplex nested PCR with primers specific to the 5S and 23S rRNA gene fragments flanking the intergenic spacer of *B. burgdorferi* (*s.l*.) and to the *glpQ* gene of *B. miyamotoi,* which were designed previously [14, 44, 71]. The primers NC1, NC2 and Q1, Q4 were used for primary reactions, while primers NC3, NC4 and Q2, Q3 were used for nested reactions (Table 2). The length of the nested PCR products was 246–253 bp for *B. burgdorferi* (*s.l*.) and 424 bp for *B. miyamotoi.*

To identify bacteria species from the *B. burgdorferi* (*s.l*.) complex (with the exception of mixed *Borrelia* infection), nested PCR with primers specific to the *clpA* gene was carried out; primers clpAF1237 and clpAR2218 were used for primary reactions, and primers clpAF1255 and clpAR2104 were used for nested reactions, as described previously [72]. The length of the nested PCR products was 849 bp for all *B. burgdorferi* (*s.l*.) species. In addition, these samples were amplified using primers specific to the *p83/100* gene; primers F7 and F10 were used for primary reactions, and primers F5 and F12 were used for nested reactions (Table 2). The length of the nested PCR products was 336 bp for *B. afzelii*, 426–462 bp for *B. bavariensis* and *B. garinii,* and 420 bp for *B. valaisiana.*

All amplified *clpA* and *p83/100* gene fragments of *B. burgdorferi* (*s.l*.) and *glpQ* gene fragments of *B. miyamotoi* were sequenced. To discriminate the closely related *B. garinii* and *B. bavariensis*, the determined *clpA* gene sequences were analyzed using the MLST website (http://pubmlst.org/borrelia/), while the *p83/100* gene sequences were compared with corresponding sequences of *B. bavariensis* strains PBi (GenBank CP000013), NMJW1 (GenBank CP003866), and BgVir (GenBank CP003202) and *B. garinii* strains N34 (GenBank AY583360), Tom203 (GenBank DQ916329), and Tom3305 (GenBank DQ916322), all of which are available in the GenBank database.

### Detection and genotyping of *Rickettsia* spp.

For screening analysis, *Rickettsia* DNA was detected by nested PCR of the *gltA* gene using primers glt1 and glt2 for primary reactions and glt3 and glt4 for nested reactions, as described previously [49]. To identify *Rickettsia* spp. in positive samples, nested reactions were performed independently using primers RT1 and RT2, specific to “*Ca.* R. tarasevichiae”, and RH1 and RH3, specific to spotted fever group rickettsiae (SFGR) (Table 2). The amplified *gltA* gene fragments of all ticks that were positive for SFGR and some that were positive for “*Ca.* R. tarasevichiae” were sequenced.

### Detection and genotyping of *Anaplasmataceae* bacteria

Detection of *Anaplasmataceae* bacteria with subsequent species determination was conducted using nested PCR assays as described previously [45]. For screening analysis, *Anaplasmataceae* DNA was detected by nested PCR based on the 16S rRNA gene. The primers Ehr1 and Ehr2 were used for primary reactions and the primers Ehr3 and Ehr4 were used for nested reactions (Table 2); the final products were 524 bp in length. For all positive samples, nested reactions were performed with primers specific to *A. phagocytophilum*, HGE1 and HGE2, and primers specific to *E. muris*, Em1 and Em2 (Table 2). For sequence analysis, fragments of the *groESL* operon with a length of 1320–1360 bp were amplified using the primers HS1-f and HS6-r (modified HS1 and HS6 primers [73]) for the primary reactions and the primers HS3-f and HSVR [74] for nested reactions.

### Detection and genotyping of *Babesia* spp.

*Babesia* DNA was detected by nested PCR for the presence of the 18S rRNA gene, as described previously [46]. Primary reactions were carried out using the forward primer BS1 and the reverse primer BS2. Nested reactions were carried out as multiplex reactions using the forward primers BS3 and BS5 and the reverse primer BS4 (Table 2). The BS3 primer was specific for the *Bab. microti* group, while the BS5 primer was specific for the *Babesia* (*sensu stricto*) group. All amplified *Babesia* spp. 18S rRNA gene fragments were sequenced.

### Sequencing and phylogenetic analysis

The PCR products were purified using GeneJET Gel Extraction Kit (ThermoFisher Scientific, Vilnius, Lithuania). The Sanger sequencing reactions were conducted using “BigDye™ Terminator v. 3.1 Cycle Sequencing kit” (Applied Biosystems Inc., Austin, TX, USA) in both directions with primers indicated in Table 2. The corresponding products were analyzed using an ABI 3500 Genetic Analyzer (Applied Biosystems Inc.). All obtained sequences were compared with those of reference strains available in the NCBI website using the BLASTN 2.2.31+ (https://blast.ncbi.nlm.nih.gov/Blast.cgi). Molecular phylogenetic analyses were conducted using Maximum Likelihood (ML) method based on Hasegawa-Kishino-Yano (HKY) nucleotide substitution model in MEGA 7.0 with 1000 bootstrap replicates [75].

### Statistical analysis

Statistical analysis was performed to compare the proportion of collected *Ixodes* spp. from various locations and prevalence of causative agents in different tick species. The 95% confidence intervals (CI) for the prevalence of infectious agents in questing ticks were computed using an Excel spreadsheet (http://www.pedro.org.au/english/downloads/confidence-interval-calculator/). Differences in the prevalence of infectious agents in *I. pavlovskyi* and *I. persulcatus* ticks per region were computed using the Pearson *χ* ^2^ goodness-of-fit test (http://www.socscistatistics.com/tests/chisquare/). *P* < 0.05 was regarded as significant.

### Nucleotide sequence accession numbers

Nucleotide sequences determined in this study were deposited in the GenBank database under the following accession numbers: KY002831–KY002882, for TBEV; KX834332, KX834343, KX834344 and KX834341 for KEMV; KX980208–KX980214 (*clpA*) and KX980275–KX980287 (*p83/100*) for *B. afzelii*; KX980215–KX980232 (*clpA*) and KX980288–KX980313 (*p83/100*) for *B. bavariensis*; KX980233–KX980273 (*clpA*) and KX980314–KX980349 (*p83/100*) for *B. garinii*; KX980274 (*clpA*) and KX980350 (*p83/100*) for *B. valaisiana*; KY006159–KY006162 for *B. miyamotoi*; KX963401–KX963404 for *R. heilongjiangensis*; KX963385–KX963388 for *R. helvetica*; KX963389–KX963395, KY019068 and KY056616–KY056618 for *R. raoultii*; KX963396–KX963400 and KY019069 for *R. sibirica*; KX963381–KX963384 for “*Ca.* R. tarasevichiae”; KX963405–KX963406 for *Rickettsia* spp.; KX980041–KX980046 for *A. phagocytophilum*; KX980047–KX980049 for *E. muris*; KX980039–KX980040 for “*Ca*. N. mikurensis”; KX987863 and KX987864 for *Bab. microti*.

## Results

### Tick species determination

Adult questing *Ixodes* spp. ticks were collected by flagging in two regions within the Western Siberian part of the *I. pavlovskyi* distribution area. Sites A1 and A2 were located in the northern part of the Altai Mountains (Republic of Altai), within the previously known distribution area of this species, while sites N1-N5 were located more northward, in the Western Siberia Plain (Novosibirsk Province), a recently invaded habitat of *I. pavlovskyi* ticks (Fig. 1). Sites A1 and A2 were located on mountain slopes with relatively low human influence, while Sites N1-N5 were located in parks and suburban areas of the city of Novosibirsk and are characterized by a substantial anthropogenic impact. A total of 2049 adult *Ixodes* ticks were collected in all sites. Of these, 1053 individuals were caught in the Altai Mountains and 996 in the Novosibirsk Province (Table 1).

Tick species were determined using morphological keys and two genetic loci, the mitochondrial *cox*1 gene and the nuclear ITS2. According to their morphology, 784 ticks collected from both regions were identified as *I. pavlovskyi* and 1156 as *I. persulcatus* (Table 1)*.* Then, all morphologically identified *I. pavlovskyi* ticks and 522 *I. persulcatus* ticks were tested genetically (only 189 of the 823 ticks collected at Site A2 and defined as *I. persulcatus* using morphological keys were subjected to further genetic analysis) (Table 1). Only ticks with both morphological and genetic criteria corresponding to the same tick species were identified as that species. All morphological intermediates and ticks with a mitochondrial locus belonging to one species and a nuclear locus to another species were excluded from this investigation. Therefore, 577 *I. pavlovskyi* and 334 *I. persulcatus* ticks that complied with both the morphological and genetic criteria were examined for the presence of tick-transmitted agents (Tables 1 and 3).

**Table 3:** Detection of tick-transmitted agents in *I. pavlovskyi* and *I. persulcatus* ticks

Region (Year)	Site	Tick species	No. of ticks from the site	No./% of ticks infected by any of tested agent	No./% of ticks containing nucleic acids of tested agents^a^								
					TBEV	KEMV	B.burg.(*s.l*.)	B.miyam	Rick.spp.	A.phag	E.mur	“Ca.N.m”	Bab.m
Alt (2012, 2014, 2015)	A1,	*I. pavl*	11	5/45.5	0	0	4/36.4	1/9.1	0	1/9.1	1/9.1	0	0
		*I. pers*	33	31/93.9	0	2/6.1	12/36.4	0	26/78.8	6/18.2	2/6.1	0	0
	A2	*I. pavl*	113	73/64.6	12/10.6	1/0.9	54/47.8	8/7.1	11/9.7	2/1.8	0	1/0.9	2/1.8
		*I. pers*	152	142/93.4	7/4.6	0	58/38.2	11/7.2	136/89.5	9/5.9	25/16.4	0	0
	Total A1-A2	*I. pavl*	124	78/62.9	12/9.7	1/0.8	58/46.8	9/7.3	11/8.9	3/2.4	1/0.8	1/0.8	2/1.6
		*I. pers*	185	173/93.5	7/3.8	2/1.1	70/37.8	11/5.9	162/87.6	15/8.1	27/14.6	0	0
Nov (2010, 2014, 2015)	N1	*I. pavl*	316	178/56.3	15/4.7	1/0.3	134/42.4	21/6.6	14/4.4	14/4.4	0	2/0.6	0
		*I. pers*	22	20/90.9	2/9.1	0	6/27.3	3/13.6	17/77.3	0	0	0	0
	N2	*I. pavl*	22	17/77.3	0	0	5/22.7	3/13.6	14/63.6	0	0	3/13.6	0
		*I. pers*	0	–	–	–	–	–	–	–	–	–	–
	N3	*I. pavl*	79	47/59.5	6/7.6	0	38/48.1	4/5.1	3/3.8	2/2.5	1/1.3	1/1.3	0
		*I. pers*	110	89/80.9	8/7.3	0	46/41.8	6/5.5	70/63.6	6/5.5	12/10.9	2/1.8	2/1.8
	N4	*I. pavl*	22	9/40.9	0	0	9/40.9	0	1/4.5	0	0	2/9.1	0
		*I. pers*	14	10/71.4	1/7.1	0	3/21.4	1/7.1	9/62.3	0	1/7.1	0	0
	N5	*I. pavl*	14	7/50.0	1/7.1	0	2/14.3	0	4/28.6	0	0	0	0
		*I. pers*	3	1/33.3	0	0	1/33.3	0	1/33.3	0	0	0	0
	Total N1-N5	*I. pavl*	453	258/57.0	22/4.9	1/0.2	188/41.5	28/6.2	36/7.9	16/3.5	1/0.2	8/1.8	0
		*I. pers*	149	120/80.5	11/7.4	0	56/37.6	10/6.7	97/65.1	6/4.0	13/8.7	2/1.3	2/1.3
Both regions		*I. pavl*	577	336/58.2	34/5.9	2/0.3	246/42.6	37/6.4	47/8.1	19/3.3	2/0.3	9/1.6	2/0.3
		*I. pers*	334	293/87.7	18/5.4	2/0.6	126/37.7	21/6.3	259/77.5	21/6.3	40/12.0	2/0.6	2/0.6

*Abbreviations*: *Alt* Republic of Altai, *Nov* Novosibirsk Province, *I. pavl I. pavlovskyi*, *I. pers I. persulcatus*, *B.burg*.(*s.l*.) *B. burgdorferi* (*s.l*.), *B.miyam B. miyamotoi*, *Rick.spp*. *Rickettsia* spp., *A.phag A. phagocytophilum*, *E.mur E. muris*, *“Ca.N.m”* “*Ca*. N. mikurensis”, *Bab.m Bab. microti*

^a^Including cases of mixed infection

*Ixodes pavlovskyi* ticks were identified in all studied sites from both the Altai Mountains and Novosibirsk surroundings. The proportion of this tick species in the Altai Mountains was 11.8% (124/1053), varying from 11.3 to 20% in different sites (Table 1). In the suburbs of Novosibirsk, the proportion of *I. pavlovskyi* ticks was 48.5% (453/996) and ranged from 18.2 to 81.5% in different sites, which was significantly higher (*χ* ^2^ = 287.450, *df* = 1, *P* < 0.001) than in the Altai Mountains. *Ixodes persulcatus* ticks were detected in almost all sites, with the exception of Site N2 from Novosibirsk Province, in which only 27 ticks were caught. In all other sites from the suburbs of Novosibirsk, the proportion of *I. persulcatus* ticks was 15.4% (149/996), varying from 4.9 to 25.4% in different sites (Table 1), which was a significantly lower proportion than that observed for *I. pavlovskyi* (*χ* ^2^ = 220.001, *df* = 1, *P* < 0.001).

### Detection and genotyping of TBEV and KEMV RNA

**Fig. 2:**
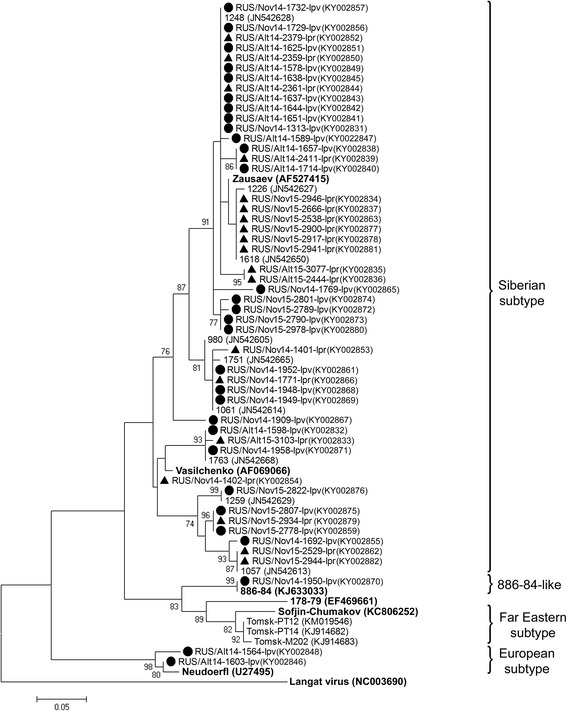
The phylogenetic tree constructed by the ML method based on nucleotide sequences of 211 bp fragment of the E gene of TBEV. The *scale-bar* indicates an evolutionary distance of 0.05 nucleotides per position in the sequence. Significant *bootstrap values* (>70%) are shown on the nodes. The sequences of prototype TBEV strains and outgroup virus (Langat virus) from GenBank database are in *boldface. Legend*: ● *I. pavlovskyi* ticks; ▲ *I. persulcatus* ticks

The TBEV prevalence in *I. pavlovskyi* and *I. persulcatus* ticks collected in both the Altai Mountains and Novosibirsk suburbs was 5.9% (34/577; 95% CI: 4.3–8.1) and 5.4% (18/334; 95% CI: 3.4–8.4), respectively (Table 3), which was not significantly different (*χ* ^2^ = 0.100, *df* = 1, *P* = 0.752) (Table 4). The sequences of TBEV isolates from most of the *I. pavlovskyi* and *I. persulcatus* ticks collected in both the Republic of Altai and Novosibirsk Province were related to each other and belonged to both the Vasilchenko and Zausaev lineages of the Siberian subtype. Among those belonging to the Zausaev lineage, several isolates detected in *I. pavlovskyi* ticks from Novosibirsk Province (KY002872–KY002874, KY002880) formed a separate cluster on the phylogenetic tree (Fig. 2). In addition, two TBEV isolates belonging to the European subtype were discovered in *I. pavlovskyi* ticks (KY002846, KY002848), which is the first reported finding of this subtype in this tick species (Fig. 2). Moreover, one TBEV isolate detected in an *I. pavlovskyi* tick (KY002870) collected from Site N1 (Novosibirsk Province) belonged to a putative new TBEV subtype currently named “886–84”, which was recently discovered in *I. persulcatus* ticks and small mammals in the Baikal region as well as in a brain sample of a deceased human in Mongolia [33, 34, 76]. Therefore, this is the first observation of the 886–84 subtype both in *I. pavlovskyi* ticks and in Western Siberia.

**Table 4:** Overall prevalence of tick-transmitted agents in *I. pavlovskyi* and *I. persulcatus* ticks per region

Region	*I. pavlovskyi* % (pos/total)	95% CI	*I. persulcatus* % (pos/total)	95% CI	*χ* ^2^	*P*
TBEV						
Alt	9.7 (12/124)	5.6–16.2	3.8 (7/185)	1.8–7.6	4.469	0.035
Nov	4.9 (22/453)	3.2–7.2	7.4 (11/149)	4.2–12.7	1.381	0.240
Total	5.9 (34/577)	4.3–8.1	5.4 (18/334)	3.4–8.4	0.100	0.752
KEMV						
Alt	0.8 (1/124)	0.1–4.4	1.1 (2/185)	0.3–3.9	0.054	0.816
Nov	0.2 (1/453)	0.0–1.2	0 (0/149)	–	0.330	0.566
Total	0.3 (2/577)	0.1–1.3	0.6 (2/334)	0.2–2.2	0.308	0.579
*B. afzelii*						
Alt	2.4 (3/124)	0.8–6.9	10.3 (19/185)	6.7–15.5	6.920	0.009
Nov	1.1 (5/453)	0.5–2.6	13.4 (20/149)	8.9–19.8	42.749	<0.001
Total	1.4 (8/577)	0.7–2.7	11.7 (39/334)	8.7–15.6	45.780	<0.001
*B. bavariensis*						
Alt	0 (0/124)	–	27.0 (50/185)	21.1–33.9	39.983	<0.001
Nov	1.3 (6/453)	0.6–2.9	18.8 (28/149)	13.3–25.8	64.197	<0.001
Total	1.0 (6/577)	0.5–2.3	23.4 (78/334)	19.1–28.2	125.853	<0.001
*B. garinii*						
Alt	45.2 (56/124)	36.7–53.9	2.7 (5/185)	1.2–6.2	84.470	<0.001
Nov	39.3 (178/453)	34.9–43.9	8.7 (13/149)	5.2–14.4	48.368	<0.001
Total	40.6 (234/577)	38.2–46.2	5.4 (18/334)	3.4–8.4	130.733	<0.001
*B. valaisiana*						
Alt	0 (0/124)	–	0 (0/185)	–	-	-
Nov	0 (0/453)	–	0.7 (1/149)	0.1–3.7	3.045	0.081
Total	0 (0/577)	–	0.3 (1/334)	0.1–1.7	1.724	0.189
All *B. burgdorferi* (*s.l.*)						
Alt	46.8 (58/124)	38.2–55.5	37.8 (70/185)	31.2–45.0	2.443	0. 118
Nov	41.5 (188/453)	37.1–46.1	37.6 (56/149)	30.0–45.6	0.714	0. 398
Total	42.6 (246/577)	38.7–46.7	37.7 (126/334)	32.7–43.0	2.111	0. 146
*B. miyamotoi*						
Alt	7.3 (9/124)	3.9–13.2	5.9 (11/185)	3.4–10.3	0.211	0.646
Nov	6.2 (28/453)	4.3–8.8	6.7 (10/149)	3.7–11.9	0.053	0.817
Total	6.4 (37/577)	4.7–8.7	6.3 (21/334)	4.2–9.4	0.006	0.941
*R. heilongjiangensis*						
Alt	0.8 (1/124)	0.1–4.4	0.5 (1/185)	0.1–3.0	0.082	0.775
Nov	0.9 (4/453)	0.3–2.3	0 (0/149)	–	1.325	0.250
Total	0.9 (5/577)	0.4–2.0	0.3 (1/334)	0.1–1.7	1.040	0.308
*R. helvetica*						
Alt	8.1 (10/124)	4.4–14.2	0 (0/185)	–	15.418	<0.001
Nov	2.2 (10/453)	1.2–4.0	0.7 (1/149)	0.1–3.7	1.475	0.225
Total	3.5 (20/577)	2.3–5.3	0.3 (1/334)	0.1–1.7	9.420	0.002
*R. raoultii*						
Alt	0 (0/124)	–	7.0 (13/185)	4.2–11.7	9.096	0.003
Nov	3.3 (15/453)	1.2–4.0	4.7 (7/149)	2.3–9.4	0.612	0.434
Total	2.6 (15/577)	1.6–4.2	6.0 (20/334)	3.9–9.1	6.574	0.010
*R. sibirica*						
Alt	0 (0/124)	–	3.2 (6/185)	1.5–6.7	4.101	0.043
Nov	0 (0/453)	–	1.3 (2/149)	0.4–4.8	6.101	0.014
Total	0 (0/577)	–	2.4 (8/334)	1.2–4.7	13.942	<0.001
“*Ca.* R. tarasevichiae”						
Alt	0.8 (1/124)	0.1–4.4	87.0 (161/185)	81.4–91.1	221.280	<0.001
Nov	2.0 (9/453)	1.0–3.7	61.7 (92/149)	53.7–69.2	286.759	<0.001
Total	1.7 (10/577)	0.9–3.2	75.7(253/334)	70.1–80.0	564.357	<0.001
All *Rickettsia* spp.						
Alt	8.9 (11/124)	5.0–15.2	87.6 (162/185)	82.0–91.6	186.586	<0.001
Nov	7.9 (36/453)	5.8–10.8	65.1 (97/149)	57.2–75.3	212.787	<0.001
Total	8.1 (47/577)	6.2–10.7	77.5 (259/334)	72.8–81.7	456.746	<0.001
*A. phagocytophilum*						
Alt	2.4 (3/124)	0.8–6.9	8.1 (15/185)	5.0–13.0	4.380	0.036
Nov	3.5 (16/453)	2.2–5.7	4.0 (6/149)	1.9–8.5	0.078	0.780
Total	3.3 (19/577)	2.1–5.1	6.3 (21/334)	4.2–9.4	4.519	0.034
*E. muris*						
Alt	0.8 (1/124)	0.1–4.4	14.6 (27/185)	10.2–20.4	17.128	<0.001
Nov	0.2 (1/453)	0.0–1.2	8.7 (13/149)	5.2–14.4	35.692	<0.001
Total	0.3 (2/577)	0.1–1.3	12.0 (40/334)	8.9–15.9	65.056	<0.001
“*Ca*. N. mikurensis”						
Alt	0.8 (1/124)	0.1–4.4	0 (0/185)	–	1.497	0.221
Nov	1.8 (8/453)	0.9–3.5	1.3 (2/149)	0.4–4.8	0.123	0.726
Total	1.6 (9/577)	0.8–2.9	0.6 (2/334)	0.2–2.2	1.637	0.201
*Bab. microti*						
Alt	1.6 (2/124)	0.4–5.7	0 (0/185)	–	3.003	0.083
Nov	0 (0/453)	–	1.3 (2/149)	0.4–4.8	6.101	0.014
Total	0.3 (2/577)	0.1–1.3	0.6 (2/334)	0.2–2.2	0.308	0.579

*Abbreviations*: *Alt* Republic of Altai, *Nov* Novosibirsk Province, *pos/total* infected ticks/examined ticks

**Fig. 3:**
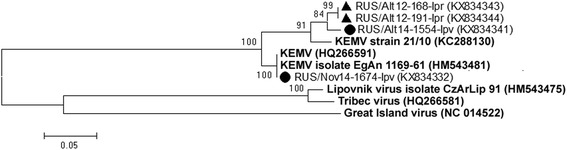
The phylogenetic tree constructed by the ML method based on nucleotide sequences of 238 bp fragment of KEMV genome segment 1. The *scale-bar* indicates an evolutionary distance of 0.05 nucleotides per position in the sequence. Significant *bootstrap values* (>70%) are shown on the nodes. The sequences of prototype KEMV strains and outgroup viruses (Great Island, Tribec and Lipovnik viruses) from GenBank database are in *boldface. Legend*: ● *I. pavlovskyi* ticks; ▲ *I. persulcatus* ticks

RNA of KEMV was found in two *I. pavlovskyi* ticks from both the Republic of Altai (Site A2) and Novosibirsk Province (Site N1) as well as two *I. persulcatus* ticks from the Republic of Altai (Site A1) (Table 3). Based on the segment 1 fragment sequence, one KEMV isolate from the *I. pavlovskyi* tick caught in the Novosibirsk suburbs (KX834332) differed from two corresponding KEMV sequences available in the GenBank database, one belonging to strain EgAn 1169–61 (GenBank HM543481), isolated in Egypt, and the other to strain 21/10 (GenBank KC288130), isolated from an *I. persulcatus* tick from Kemerovo Province in Western Siberia (identity level of 93.2 and 92.4%, respectively) (Fig. 3). However, three other KEMV isolates collected in the Altai Mountains, the two isolates from *I. persulcatus* (KX834343 and KX834344) and one from *I. pavlovskyi* (KX834341) ticks, were more closely related to KEMV strain 21/10 (identity level of 95.7–95.8%).

### Detection and genotyping of *Borrelia* spp. spirochetes

*Borrelia burgdorferi* (*s.l*.) DNA was found in 42.6% (246/577; 95% CI: 38.7–46.7) of *I. pavlovskyi* ticks and 37.7% (126/334; 95% CI: 32.7–43.0%) of the examined *I. persulcatus* ticks (Tables 3 and 4). The prevalence of *B. burgdorferi* (*s.l*.) in *I. pavlovskyi* ticks from the Republic of Altai and Novosibirsk Province did not significantly differ (46.8%; 95% CI: 38.2–55.5 and 41.5%; 95% CI: 37.1–46.1, respectively) and was similar to that of *I. persulcatus* ticks from the same regions (37.8%; 95% CI: 31.2–45.0 and 37.6%; 95% CI: 30.0–45.6, respectively). In two of the Novosibirsk sites (sites N2 and N5), the prevalence rates of *B. burgdorferi* (*s.l*.) in *I. pavlovskyi* ticks were lower, 22.7 and 14.3%, respectively, than in other sites (Table 3). However, the samples from these sites were small.

Among spirochetes of the *B. burgdorferi* (*s.l*.) complex, four *Borrelia* species were identified in this study: *B. afzelii*, *B. bavariensis*, *B. garinii* and *B. valaisiana* (Table 5). In total, including cases of mixed infection, *B. afzelii* was detected in 1.4% (8/577; 95% CI: 0.7–2.7) of *I. pavlovskyi* ticks and in 11.7% (39/334; 95% CI: 8.7–15.6) of *I. persulcatus* ticks; *B. bavariensis* was revealed in 1.0% (6/577; 95% CI: 0.5–2.3) of *I. pavlovskyi* ticks and in 23.4% (78/334; 95% CI: 19.1–28.2) of *I. persulcatus* ticks; *B. garinii* was found in 40.6% (234/577; 95% CI: 38.2–46.2) of *I. pavlovskyi* ticks and in 5.4% (18/334; 95% CI: 3.4–8.4) of *I. persulcatus* ticks (Table 4). Notably, this was the first discovery of *B. bavariensis* in *I. pavlovskyi* ticks, and all cases were recorded in Novosibirsk Province. Apparently, *B. afzelii* and *B. bavariensis* were detected significantly less often (*χ* ^2^ = 45.780, *df* = 1, *P* < 0.001 and *χ* ^2^ = 125.853, *df* = 1, *P* < 0.001, respectively) in *I. pavlovskyi* ticks than in *I. persulcatus* ticks (Tables 4 and 5). By contrast, the prevalence of *B. garinii* in *I. pavlovskyi* ticks collected in the Republic of Altai and Novosibirsk Province (45.2%; 95% CI: 36.7–53.9 and 39.3%; 95% CI: 34.9–43.9, respectively) was significantly higher (*χ* ^2^ = 39.983, *df* = 1, *P* < 0.001, for Altai, and *χ* ^2^ = 64.197, *df* = 1, *P* < 0.001, for Novosibirsk Province) than that in *I. persulcatus* ticks caught in the same regions (2.7%; 95% CI: 1.2–6.2 and 8.7%; 95% CI: 5.2–14.4, respectively) (Table 4). *Borrelia valaisiana* was not found in *I. pavlovskyi* ticks and was detected in only one *I. persulcatus* tick caught near Novosibirsk, which is the first finding of this bacterium in Novosibirsk Province (Table 5).

**Table 5:** Detection of *Borrelia* spp. in *I. pavlovskyi* and *I. persulcatus* ticks

Region (Year)	Sites	Tick species	No. of examined ticks	No./% of ticks containing DNA of									
				All *Borrelia* spp.	Ba	Bb	Bg	Bv	Ba + Bb	Ba + Bg	Bb + Bg	Bm	Bg + Bm
Alt (2012, 2014, 2015)	A1	*I. pavl*	11	4/36.4	0	0	3/27.3	0	0	0	0	0	1/9.1
		*I. pers*	33	12/36.4	7/21.2	4/12.1	1/3.0	0	0	0	0	0	0
	A2	*I. pavl*	113	62/54.9	2/1.8	0	51/45.1	0	0	1/0.9	0	8/7.1	0
		*I. pers*	152	69/45.4	8/5.3	42/27.6	4/2.6	0	4/2.6	0	0	11/7.2	0
	Total A1-A2	*I. pavl*	124	66/53.2	2/1.6	0	54/43.5	0	0	1/0.8	0	8/6.5	1/0.8
		*I. pers*	185	81/43.8	15/8.1	46/24.9	5/2.7	0	4/2.2	0	0	11/5.9	0
Nov (2010, 2014, 2015)	N1	*I. pavl*	316	152/48.1	1/0.3	1/0.3	129/40.8	0	0	0	0	19/6.0	2/0.6
		*I. pers*	22	9/40.9	0	3/13.6	3/13.6	0	0	0	0	3/13.6	0
	N2	*I. pavl*	22	8/36.4	0	0	5/22.7	0	0	0	0	3/13.6	0
		*I. pers*	0	–	–	–	–	–	–	–	–	–	–
	N3	*I. pavl*	79	42/53.2	1/1.3	2/2.5	33/41.8	0	0	1/1.3	1/1.3	4/5.1	0
		*I. pers*	110	52/47.3	13/11.8	17/15.5	9/8.2	1/0.9	6/5.5	0	0	6/5.5	0
	N4	*I. pavl*	22	9/40.9	2/9.1	2/9.1	5/22.7	0	0	0	0	0	0
		*I. pers*	14	4/28.6	1/7.1	1/7.1	1/7.1	0	0	0	0	1/7.1	0
	N5	*I. pavl*	14	2/14.3	0	0	2/14.3	0	0	0	0	0	0
		*I. pers*	3	1/33.3	0	1/33.3	0	0	0	0	0	0	0
	Total N1-N5	*I. pavl*	453	213/47.0	4/0.9	5/1.1	174/38.4	0	0	1/0.2	1/0.2	26/5.7	2/0.4
		*I. pers*	149	66/44.3	14/9.4	22/14.8	13/8.7	1/0.7	6/4.0	0	0	10/6.7	0
Both regions		*I. pavl*	577	279/48.4	6/1.0	5/0.9	228/39.5	0	0	2/0.3	1/0.2	34/5.9	3/0.5
		*I. pers*	334	147/44.0	29/8.7	68/20.4	18/5.4	1/0.3	10/3.0	0	0	21/6.3	0

*Abbreviations*: *Alt* Republic of Altai, *Nov* Novosibirsk Province, *I. pavl I. pavlovskyi*, *I. pers I. persulcatus*, *Ba B. afzelii*, *Bb B. bavariensis*, *Bg B. garinii*, *Bv B. valaisiana*, *Bm B. miyamotoi*

**Fig. 4:**
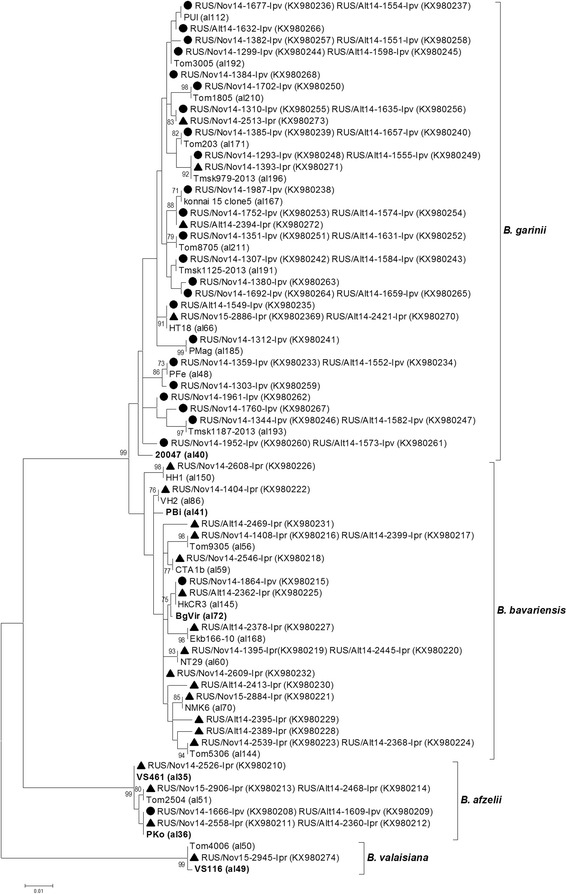
The phylogenetic tree constructed by the ML method based on nucleotide sequences of 579 bp fragment of the *clpA* gene of *Borrelia* spp. from *Borrelia burgdorferi* (*s.l*.) complex. The *scale-bar* indicates an evolutionary distance of 0.01 nucleotides per position in the sequence. Significant *bootstrap values* (>70%) are shown on the nodes. The sequences of prototype strains of *Borrelia* spp. from the *Borrelia* MLST website are in *boldface. Legend*: ● *I. pavlovskyi* ticks; ▲ *I. persulcatus* ticks

The determined *clpA* gene sequences of *B. afzelii* found in both *I. pavlovskyi* and *I. persulcatus* ticks (KX980208–KX980214) were identical to known alleles deposited in the *Borrelia* MLST website (http://pubmlst.org/borrelia/) (Fig. 4). Only known *clpA* gene allele (KX980215) belonging to *B. bavariensis* was found in *I. pavlovskyi* ticks, while five new (KX980228–KX980232) and nine known (KX980216–KX980227) alleles were recorded in *I. persulcatus* ticks from both the Altai and Novosibirsk regions. Nine new *clpA* gene alleles of *B. garinii* were found in *I. pavlovskyi* ticks (KX980257–KX980268) and two new *clpA* gene alleles were identified in both *I. pavlovskyi* and *I. persulcatus* ticks (KX980253–KX980256, KX980272–KX980273). In addition, ten known *clpA* gene alleles of *B. garinii* were recorded in *I. pavlovskyi* ticks (KX980236–KX980252), and two known *clpA* gene alleles of *B. garinii* were found in both *I. pavlovskyi* and *I. persulcatus* ticks (KX980233–KX980235, KX980269–KX980271). A single *clpA* gene sequence belonging to *B. valaisiana* (KX980274) was identical to a previously published sequence (Fig. 4) that was detected in an *I. persulcatus* tick caught in Tomsk Province (Western Siberia) [77].

**Fig. 5:**
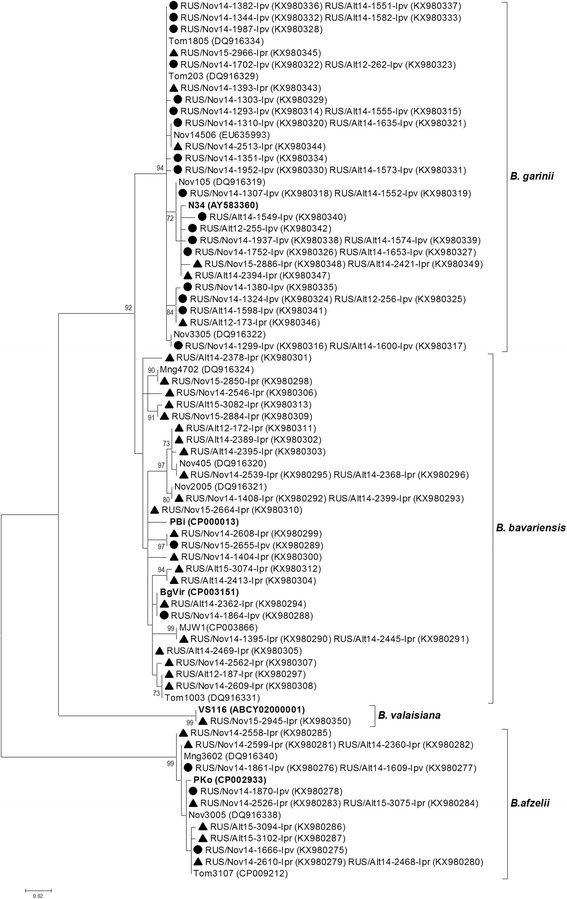
The phylogenetic tree constructed by the ML method based on nucleotide sequences of 276–402 bp fragment of the *p83/100* gene of *Borrelia* spp. from *Borrelia burgdorferi* (*s.l*.) complex. The *scale-bar* indicates an evolutionary distance of 0.02 nucleotides per position in the sequence. Significant *bootstrap values* (>70%) are shown on the nodes. The sequences of prototype strains of *Borrelia* spp. from GenBank database are in *boldface. Legend*: ● *I. pavlovskyi* ticks; ▲ *I. persulcatus* ticks

Analysis of the sequenced *p83/100* gene fragments of *B. burgdorferi* (*s.l*.) revealed six genetic variants of *B. afzelii*, 21 genetic variants of *B. bavariensis,* and 19 genetic variants of *B. garinii* (Fig. 5). Among them, three variants of *B. afzelii* (KX980285–KX980287), 15 variants of *B. bavariensis* (KX980289, KX980299–KX980313), and 14 variants of *B. garinii* (KX980324–KX980342, KX980346–KX980349) were new, while the other genetic variants were previously observed in *I. persulcatus* ticks [78]. Of the new genetic variants, 11 variants of *B. garinii* (KX980328–KX980342) were identified in *I. pavlovskyi* ticks (both in the Altai and Novosibirsk regions); while three variants of *B. afzelii* (KX980285–KX980287), 14 variants of *B. bavariensis* (KX980300–KX980313), and one variant of *B. garinii* (KX980348–KX980349) were identified in *I. persulcatus* ticks. In addition, a new variant of *B. bavariensis* (KX980289, KX980299) and two new variants of *B. garinii* (KX980324–KX980327, KX980346–KX980347) were identified in both tick species. Notably, one new *p83/100* genetic variant belonging to *B. garinii* that was only found in *I. pavlovskyi* ticks collected in both the Republic of Altai and Novosibirsk Province (KX980338–KX980339) was unusual and included a 36 bp insertion. In addition, a genetic variant of the *B. bavariensis* p83/100 gene with a 3 bp insertion (KX980306) was identified in one *I. persulcatus* tick from Novosibirsk Province (Fig. 5).

**Fig. 6:**
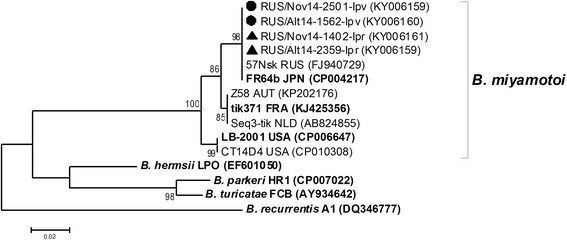
The phylogenetic tree constructed by the ML method based on nucleotide sequences of 359 bp fragment of the *glpQ* gene of *Borrelia* spp. from relapsing fever group. The *scale-bar* indicates an evolutionary distance of 0.02 nucleotides per position in the sequence. Significant *bootstrap values* (>70%) are shown on the nodes. The sequences of prototype strains of *Borrelia* spp. from GenBank database are in *boldface. Legend*: ● *I. pavlovskyi* ticks; ▲ *I. persulcatus* ticks

The prevalence of *B. miyamotoi* in *I. pavlovskyi* and *I. persulcatus* ticks collected both in the Republic of Altai and Novosibirsk Province was similar, 6.4% (37/577; 95% CI: 4.7–8.7) and 6.3% (21/334; 95% CI: 4.2–9.4), respectively. The sequences of all determined *glpQ* gene fragments of *B. miyamotoi* detected in *I. pavlovskyi* and *I. persulcatus* ticks from both regions (KY006159–KY006162) were identical to each other and to a corresponding sequence of Asian-type *B. miyamotoi* [79], which was previously identified in *I. persulcatus* ticks from Novosibirsk Province (FJ940729) (Fig. 6).

### Detection and genotyping of *Rickettsia* spp.

*Rickettsia* spp. were found in 8.1% (47/577; 95% CI: 6.2–10.7) of the examined *I. pavlovskyi* ticks and 77.5% (259/334; 95% CI: 72.8–81.7) of *I. persulcatus* ticks (Tables 3 and 4). In both regions, *I. pavlovskyi* ticks were infected with the *Rickettsia* spp. significantly less often (8.9 and 7.9% of infected individuals collected in the Republic of Altai and Novosibirsk suburbs, respectively) than *I. persulcatus*, in which the DNA of *Rickettsia* spp. was detected in 87.6% of the samples from Altai and in 65.1% of the samples from Novosibirsk Province (*χ* ^2^ = 456.746, *df* = 1, *P* < 0.001) (Table 4). Significant differences were observed in all studied sites with the exception of Sites N2 and N5, where the total number of collected *Ixodes* ticks was low.

Five *Rickettsia* species were identified and confirmed by sequencing: *R. heilongjiangensis*, *R. helvetica*, *R. raoultii*, *R. sibirica* and “*Ca.* R. tarasevichiae”, as well as two *Rickettsia* genetic variants that had not been previously found (Table 6). Notably, this is the first report of *R. heilongjiangensis*, *R. helvetica*, *R. raoultii* and “*Ca.* R. tarasevichiae” detection in *I. pavlovskyi* ticks. Distribution of several *Rickettsia* species varied between *I. pavlovskyi* and *I. persulcatus* ticks. Including cases of mixed infection, *R. heilongjiangensis* was found in 0.9% (5/577; 95% CI: 0.4–2.0) of *I. pavlovskyi* ticks and 0.3% (1/334; 95% CI: 0.1–1.7) of *I. persulcatus* ticks; *R. helvetica* was detected in 3.5% (20/577; 95% CI: 2.3–5.3) of *I. pavlovskyi* ticks and in 0.3% (1/334; 95% CI: 0.1–1.7) of *I. persulcatus* ticks; *R. raoultii* was identified in 2.6% (15/577; 95% CI: 1.6–4.2) of *I. pavlovskyi* ticks and in 6.0% (20/334; 95% CI: 3.9–9.1) of *I. persulcatus* ticks; and “*Ca.* R. tarasevichiae” was recorded in 1.7% (10/577; 95% CI: 0.9–3.2) of *I. pavlovskyi* ticks and in 75.7% (253/334; 95% CI: 70.1–80.0) of *I. persulcatus* ticks (Tables 4 and 6). In addition, *R. sibirica* and new *Rickettsia* genetic variants were only found in 2.4% (8/334) and 0.6% (2/334) of *I. persulcatus* ticks, respectively. Thus, *I. pavlovskyi* ticks were significantly less often infected by “*Ca.* R. tarasevichiae” compared to *I. persulcatus* ticks (*χ* ^2^ = 564.357, *df* = 1, *P* < 0.001) (Table 4), and significant differences were observed in all studied sites, with the exception of Sites N2 and N5, where small amounts of *Ixodes* ticks were collected. In addition, *I. pavlovskyi* ticks were significantly more often infected by *R. helvetica* (*χ* ^2^ = 9.420, *df* = 1, *P* = 0.002); however, the significant difference was only observed in the Altai region (*χ* ^2^ = 15.481, *df* = 1, *P* < 0.001) and not in Novosibirsk Province. The *R. raoultii* species was not found in *I. pavlovskyi* ticks from the Altai region; however, it was detected in both *Ixodes* species in Novosibirsk Province, though the prevalence of *R. raoultii* between these two tick species did not vary significantly (*χ* ^2^ = 0.612, *df* = 1, *P* = 0.434). The prevalence of other *Rickettsia* species was too low for reliable comparisons (Tables 4 and 6).

**Table 6:** The detection of *Rickettsia* spp. in *I. pavlovskyi* and *I. persulcatus* ticks

Region (Year)	Site	Tick species	No. of examined ticks	No./% of ticks containing DNA of										
				All *Rickettsia* spp.	Rhlg	Rh	Rr	Rs	Rt	Rhlg + Rt	Rh + Rt	Rr + Rt	Rs + Rt	R.sp.+ Rt
Alt (2012, 2014, 2015)	A1	*I. pavl*	11	0	0	0	0	0	0	0	0	0	0	0
		*I. pers*	33	26/78.8	0	0	0	0	25/75.8	0	0	1/3.0	0	0
	A2	*I. pavl*	113	11/9.7	1/0.9	9/8.0	0	0	0	0	1/0.9	0	0	0
		*I. pers*	152	136/89.5	0	0	0	1/0.7	116/76.3	1/0.7	0	12/7.9	5/3.3	1/0.7
	Total A1-A2	*I. pavl*	124	11/8.9	1/0.8	9/7.3	0	0	0	0	1/0.8	0	0	0
		*I. pers*	185	162/87.6	0	0	0	1/0.5	141/76.2	1/0.5	0	13/7.0	5/2.7	1/0.5
Nov (2010, 2014, 2015)	N1	*I. pavl*	316	14/4.4	2/0.6	7/2.2	0	0	3/0.9	0	2/0.6	0	0	0
		*I. pers*	22	17/77.3	0	0	4/18.2	0	12/54.5	0	1/4.5	0	0	0
	N2	*I. pavl*	22	14/63.6	2/9.1	1/4.5	11/50.0	0	0	0	0	0	0	0
		*I. pers*	0	–	–	–	–	–	–	–	–	–	–	–
	N3	*I. pavl*	79	3/3.8	0	0	0	0	3/3.8	0	0	0	0	0
		*I. pers*	110	70/63.6	0	0	0	1/0.9	65/59.1	0	0	2/1.8	1/0.9	1/0.9
	N4	*I. pavl*	22	1/4.5	0	0	0	0	1/4.5	0	0	0	0	0
		*I. pers*	14	9/64.3	0	0	0	0	9/64.3	0	0	0	0	0
	N5	*I. pavl*	14	4/28.6	0	0	4/28.6	0	0	0	0	0	0	0
		*I. pers*	3	1/33.3	0	0	0	0	0	0	0	1/33.3	0	0
	Total N1-N5	*I. pavl*	453	36/7.9	4/0.9	8/1.8	15/3.3	0	7/1.5	0	2/0.4	0	0	0
		*I. pers*	149	97/65.1	0	0	4/2.7	1/0.7	86/57.7	0	1/0.7	3/2.0	1/0.7	1/0.7
Both regions		*I. pavl*	577	47/8.1	5/0.9	17/2.9	15/2.6	0	7/1.2	0	3/0.5	0	0	0
		*I. pers*	334	259/77.5	0	0	4/1.2	2/0.6	227/68.0	1/0.3	1/0.3	16/4.8	6/1.8	2/0.6

*Abbreviations*: *Alt* Republic of Altai, *Nov* Novosibirsk Province, *I. pavl I. pavlovskyi*, *I. pers I. persulcatus*, *Rhlg R. heilongjiangensis*, *Rh R. helvetica*, *Rr R. raoultii*, *Rs R. sibirica*, *Rt* “*Ca.* R. tarasevichiae”, *Rsp* new *Rickettsia* genovariants

**Fig. 7:**
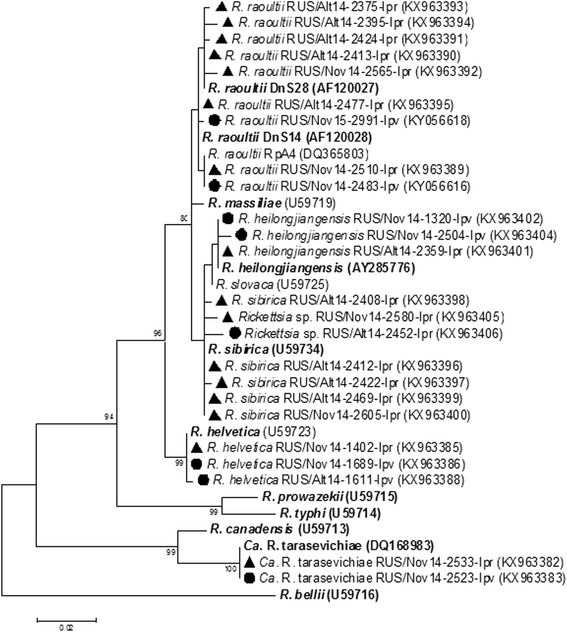
The phylogenetic tree constructed by the ML method based on nucleotide sequences of 474 bp fragment of the *gltA* gene of *Rickettsia* spp. The *scale-bar* indicates an evolutionary distance of 0.02 nucleotides per position in the sequence. Significant *bootstrap values* (>75%) are shown on the nodes. The sequences of prototype strains of *Rickettsia* spp. from GenBank database are in *boldface. Legend*: ● *I. pavlovskyi* ticks; ▲ *I. persulcatus* ticks

Most of the identified *gltA* gene sequences (Fig. 7) of *R. heilongjiangensis* (KX963401–KX963403), *R. helvetica* (KX963385–KX963387), and “*Ca.* R. tarasevichiae” (KX963381–KX963384) found in both *I. pavlovskyi* and *I. persulcatus* ticks were identical to corresponding sequences previously found in *I. persulcatus* ticks (CP002912, RHU59723 and AF503167, respectively). Only single *gltA* gene sequences of *R. heilongjiangensis* (KX963404) and *R. helvetica* (KX963388) detected in *I. pavlovskyi* ticks from the Novosibirsk Province and Altai Mountains, respectively, differed from the known corresponding sequences by 1–2 nucleotide substitutions (Fig. 7). Sequences of *gltA* gene fragments belonging to *R. raoultii* detected in *I. pavlovskyi* ticks (KY019068, KY056616, KY056617) were identical to known corresponding sequences of genetic variants previously named RpA4 and DnS14 (DQ365803 and AF120028, respectively) or differed from DnS14 by a single nucleotide substitution (KY019068, KY056618). *Rickettsia raoultii gltA* gene fragments from *I. persulcatus* ticks (KX963389–KX963395) were more variable and belonged to the RpA4, DnS14, and DnS28 genetic variants (AF120028, DQ365803 and AF120027, respectively) or differed from them by 1–3 nucleotide substitutions (KX963391–KX963394). The determined *gltA* gene sequences of *R. sibirica* found in *I. persulcatus* ticks from both the Altai and Novosibirsk regions (KX963396–KX963400, KY019069) were identical to a known sequence of *R. sibirica* (RSU59734) or differed from it by 1–2 nucleotide substitutions (KX963397, KX963398, KX963400, KY019069). The sequences of the two new *Rickettsia* spp. isolates (KX963405, KX963406) were most similar to the *R. sibirica* sequence (RSU59734), but differed from it by 4–5 nucleotide substitutions (Fig. 7).

### Detection and genotyping of *Anaplasmataceae* bacteria

Bacteria of three species belonging to the family *Anaplasmataceae* were identified in both *I. pavlovskyi* and *I. persulcatus* ticks: *A. phagocytophilum*, *E. muris* and “*Ca*. N. mikurensis”. In both the Altai and Novosibirsk regions, *A. phagocytophilum* was found in 3.3% (19/577; 95% CI: 2.1–5.1) of *I. pavlovskyi* ticks and in 6.3% (21/334; 95% CI: 4.2–9.4) of *I. persulcatus* ticks; *E. muris* was detected in 0.3% (2/577; 95% CI: 0.1–1.3) of *I. pavlovskyi* ticks and in 12.0% (40/334; 95% CI: 8.9–15.9) of *I. persulcatus* ticks; “*Ca*. N. mikurensis” was identified in 1.6% (9/577; 95% CI: 0.8–2.9) of *I. pavlovskyi* ticks and in 0.6% (2/334; 95% CI: 0.2–2.2) of *I. persulcatus* ticks (Tables 3 and 4). Thus, *A. phagocytophilum*, *E. muris* and “*Ca*. N. mikurensis” were discovered in *I. pavlovskyi* ticks for the first time, although *E. muris* DNA was detected in single individuals of this tick species, one each from the Altai and Novosibirsk regions. In both regions, *I. pavlovskyi* ticks were significantly less often infected by *E. muris* (*χ* ^2^ = 65.056, *df* = 1, *P* < 0.001) compared to *I. persulcatus* ticks (Table 4). For *A. phagocytophilum* and “*Ca*. N. mikurensis”, there was no significant difference in their prevalence between *I. pavlovskyi* and *I. persulcatus* ticks.

**Fig. 8:**
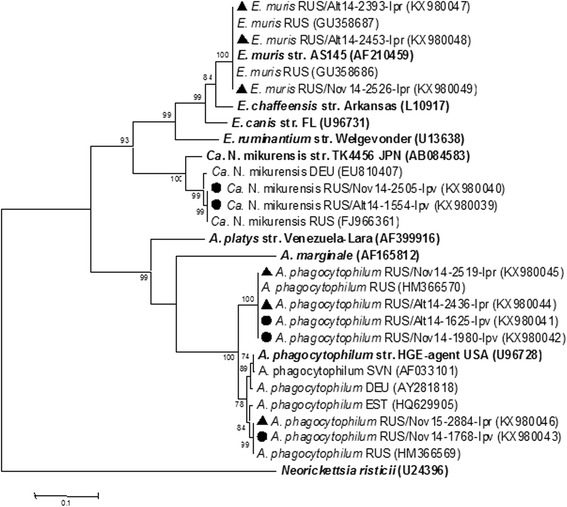
The phylogenetic tree constructed by the ML method based on nucleotide sequences of 1166–1174 bp fragment of the *groESL* operon of *Anaplasmataceae* bacteria. The *scale-bar* indicates an evolutionary distance of 0.1 nucleotides per position in the sequence. Significant *bootstrap values* (>75%) are shown on the nodes. The sequences of prototype strains of *Anaplasmataceae* bacteria from GenBank database are in *boldface. Legend*: ● *I. pavlovskyi* ticks; ▲ *I. persulcatus* ticks

Two sequence variants of the *A. phagocytophilum groESL* operon were revealed in both *I. pavlovskyi* and *I. persulcatus* ticks (KX980041–KX980043 and KX980044–KX980046, respectively); these sequences corresponded to two distinct genetic groups of *A. phagocytophilum* that were previously identified in *I. persulcatus* ticks in Russia (HM366570 and HM366569, respectively) [80] (Fig. 8). The determined *groESL* operon sequences of *E. muris* (KX980047–KX980049) corresponded to sequences that were previously found in *I. persulcatus* ticks in Russia (GU358686 and GU358687). Along with these sequences, one new *groESL* operon sequence variant (KX980048) was detected in an *I. persulcatus* tick from Altai. The “*Ca*. N. mikurensis” sequences of the *groESL* operon detected in *I. pavlovskyi* ticks were highly conserved (KX980039 and KX980040) and identical to sequences previously found in *I. persulcatus* ticks in Russia (FJ966361) (Fig. 8).

### Detection and genotyping of *Babesia* spp.

**Fig. 9:**
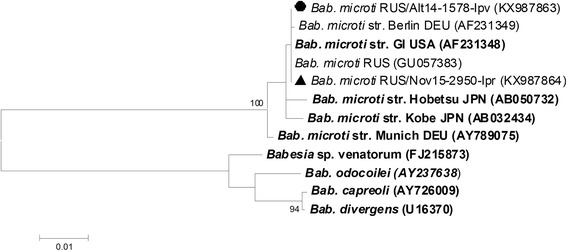
The phylogenetic tree constructed by the ML method based on nucleotide sequences of 1160–1200 bp fragment of the 18S rRNA gene of *Babesia* spp. The *scale-bar* indicates an evolutionary distance of 0.01 nucleotides per position in the sequence. Significant *bootstrap values* (>75%) are shown on the nodes. The sequences of prototype strains of *Babesia* spp. from GenBank database are in boldface. *Legend*: ● *I. pavlovskyi* ticks; ▲ *I. persulcatus* ticks

*Babesia* DNA was found only in four ticks, including two *I. pavlovskyi* ticks from the Republic of Altai and two *I. persulcatus* ticks from Novosibirsk Province (Table 3). The determined 18S rRNA gene fragment sequences of *Babesia* sp. found in both tick species (KX987863 and KX987864, respectively) were identical to corresponding sequences of US-type *Bab. microti* previously identified in *I. persulcatus* ticks collected in the Far-Eastern and Siberian parts of Russia (GU057384, GU057383) (Fig. 9). Notably, this was the first finding of *Bab. microti* in *I. pavlovskyi* ticks.

## Discussion

*Ixodes pavlovskyi* ticks are known to be closely related to *I. persulcatus* [54, 58] and, as expected, the same infectious agents could be vectored by *I. pavlovskyi*. Indeed, several human pathogens have been detected in this tick species [36, 48, 51, 66, 67]; however, thorough investigations of the ability of *I. pavlovskyi* ticks to transmit a wide range of pathogens have not been conducted. In this study, the prevalence and genetic diversity of TBEV, KEMV, *B. burgdorferi* (*s.l*.), *B. miyamotoi*, *Rickettsia* spp*.*, *Anaplasmataceae* bacteria and *Babesia* spp. were investigated in *I. pavlovskyi* ticks and compared with those in *I. persulcatus*.

*Ixodes pavlovskyi* and *I. persulcatus* were collected in two geographically distant regions where these tick species occur in sympatry: the northern part of the Altai Mountains and suburbs of Novosibirsk. Both regions are located within the Western Siberian part of the *I. pavlovskyi* disjunctive distribution area. The Altai Mountains area is the traditional habitat of this tick species, and *I. pavlovskyi* ticks have been regularly detected in this region, with the proportion ranging from 0.1 to 40% in different sites [38, 55, 58, 81]. In the suburbs of Novosibirsk, *I. pavlovskyi* ticks became more abundant only during this century and their proportion among the captured ticks varied from 3 to 94% in different locations [35, 58, 62]. Notably, the existence of *I. pavlovskyi*/*I. persulcatus* natural hybrids was previously shown [61]. To exclude possible hybrids, each adult tick was characterized using both morphological and genetic criteria, and all intermediate morphological forms and genetic crosses were excluded from this study. Unexpectedly, the occurrence of *I. pavlovskyi* ticks in the Novosibirsk suburbs was significantly higher than in the Altai Mountains, its traditional habitat area. This difference could be associated with peculiarities of the *I. pavlovskyi* and *I. persulcatus* life-cycles: adult *I. pavlovskyi* ticks feed mainly on birds, while *I. persulcatus* ticks feed on medium-sized and large mammals. Therefore, the considerable anthropogenic influence observed in the suburbs of Novosibirsk, a city of more than 1.5 million people, resulted in a substantial decrease of medium-sized and large mammals in this area that might contribute to the significantly lower *I. persulcatus* share of this tick population.

Despite the aforementioned differences in the life-cycles of *I. pavlovskyi* and *I. persulcatus* ticks, they have a similar ecology and overlapping activity periods, and their immature stages feed on the same small mammals. As a result, *I. pavlovskyi* and *I. persulcatus* larvae and nymphs can acquire the same infectious agents by feeding on infected mammals or by co-feeding with infected ticks, and these tick species can therefore transmit similar repertoires of pathogens.

Indeed, almost the full spectrum of tested infectious agents was identified in *I. pavlovskyi* ticks, including *B. bavariensis*, *R. heilongjiangensis*, *R. helvetica*, *R. raoultii*, “*Ca.* R. tarasevichiae”, *A. phagocytophilum*, *E. muris*, “*Ca.* N. mikurensis” and *Bab. microti*, which were found in this tick species for the first time (Table 3). The exceptions were *R. sibirica* and *B. valaisiana*, which were not identified in *I. pavlovskyi* ticks collected in either the Altai Mountains or the Novosibirsk suburbs. Notably, *B. valaisiana* is extremely rare in the Asian part of Russia [77, 82] and the *B. valaisiana* isolate detected in *I. persulcatus* in this study was only the second isolate found in Western Siberia. The prevalence of a number of infectious agents, including TBEV, KEMV, *B. miyamotoi*, *A. phagocytophilum*, “*Ca*. N. mikurensis”, and *Bab. microti*, was similar in both *I. pavlovskyi* and *I. persulcatus* ticks. Moreover, we could not identify specific genetic variants of these agents associated with only one of these two tick species. However, the distribution of some bacterial species belonging to the *B. burgdorferi* (*s.l*.) complex, genus *Rickettsia*, and family Anaplasmataceae significantly varied in the studied tick species.

Among spirochetes of the *B. burgdorferi* (*s.l*.) complex, *B. garinii* was significantly more often found in *I. pavlovskyi*, while *B. bavariensis* and *B. afzelii* were significantly more prevalent in *I. persulcatus* ticks (Tables 4 and 5). These data correspond to the results of other investigators, which demonstrated the presence of *B. garinii* only in *I. pavlovskyi* ticks [51]. Moreover, many new genetic variants of *B. garinii*, according to the *clpA* and *p83/100* gene sequences, were detected in *I. pavlovskyi* ticks collected from both the Altai Mountains and Novosibirsk suburbs and further investigations based on MLST are required for the genetic characterization of *Borrelia* spp. in *I. pavlovskyi* ticks. In spite of such differences in the *Borrelia* spp. distribution in these tick species, the total average prevalence of spirochetes of the *B. burgdorferi* (*s.l*.) complex in *I. pavlovskyi* and *I. persulcatus* ticks did not significantly differ in either the Altai Mountains or Novosibirsk suburbs (Table 4).

As for *Rickettsia* species, *I. pavlovskyi* ticks were shown to carry the same *Rickettsia* species as *I. persulcatus*, except *R. sibirica.* However, *I. pavlovskyi* ticks were infected with *Rickettsia* spp. significantly less often than *I. persulcatus* (8.1 *vs* 77.5%), and significant differences were observed in both the Altai Mountains and Novosibirsk suburbs (Tables 4 and 6). In *I. persulcatus* ticks, “*Ca*. R. tarasevichiae” is known to be a predominant *Rickettsia* species and was identified in 45–90% of individuals collected in most of the examined regions. *R. heilongjiangensis*, *R. helvetica*, *R. raoultii* and *R. sibirica* are substantially rarer species of *Rickettsia* [15, 40, 49, 50]. Surprisingly, *I. pavlovskyi* ticks were significantly less often infected by “*Ca*. R. tarasevichiae” (<2%) and more often infected by *R. helvetica* compared to *I. persulcatus* (Tables 4 and 6). Despite variations in the distribution of *Rickettsia* species in *I. pavlovskyi* and *I. persulcatus* ticks, most genetic variant of “*Ca*. R. tarasevichiae”, *R. helvetica* and *R. heilongjiangensis* were common for both tick species, while several new genetic variants of *R. raoultii* were found in *I. pavlovskyi* and *I. persulcatus* ticks (Fig. 7). In addition, two new genetic variants of *R. sibirica* and two new genetic variants of *Rickettsia* spp. related to *R. sibirica* were found in *I. persulcatus* ticks. Further sequencing of other genetic loci of these unusual isolates is required.

From bacteria belonging to the *Anaplasmataceae* family, monocytic *E. muris* was only detected in two *I. pavlovskyi* ticks, which was significantly rarer compared to *I. persulcatus* ticks (Tables 3 and 4). Previously, this bacterium was found in different tick species in Eurasia, including *Haemaphysalis flava*, *I. ricinus* and *I. persulcatus* [12, 15, 45, 83]. Therefore, a significant difference in its prevalence in the closely related *I. pavlovskyi* and *I. persulcatus* was unexpected.

Notably, in addition to the Siberian subtype of TBEV, which is common in these regions, a TBEV of European subtype and a putative new subtype, 886–84, were first detected in *I. pavlovskyi* ticks in this study (Fig. 2). Moreover, TBEV strains belonging to the 886–84 group (EF469662, KJ633033) were previously found only in Eastern Siberia and Mongolia [33, 76], approximately 1400–1700 km away from the suburbs of Novosibirsk, and our findings could reflect a possibly wider distribution of this genetic variant of TBEV.

Interestingly, one unusual KEMV isolate was found in an *I. pavlovskyi* tick caught in the Novosibirsk suburbs (Fig. 3). Its segment 1 fragment sequence differed considerably from the corresponding sequences of other isolates found in *Ixodes* ticks collected in the northern part of the Altai Mountains in this study, a KEMV strain isolated from *I. persulcatus* ticks from Kemerovo Province (Western Siberia, Russia) in 1968, and a KEMV strain identified from a redstart, *Phoenicurus ochruros*, in Egypt in 1961 [84]. However, the data on KEMV are limited, as only two different sequences of the KEMV segment 1 fragment are currently available in the GenBank database, and more KEMV sequences are required to interpret these results.

Thus, a number of new genetic variants of *B. garinii* and single new variants of KEMV, *R. heilongjiangensis*, *R. helvetica* and *R. raoultii* were only found in *I. pavlovskyi* ticks in this study. Previously, the association of different genetic variants of several tick-transmitted infectious agents with particular *Ixodes* ticks has been shown: specific genetic lineages of *A. phagocytophilum* were identified in *I. ricinus*, *I. persulcatus* and *I. trianguliceps* [85–87] and distinct genotypes of *Bab. microti,* US-type and Hobetsu-type, were detected in *I. persulcatus* and *I. ovatus*, respectively [19]. Conventionally, different subtypes of TBEV and types of *B. miyamotoi* have been associated with *I. ricinus* and *I. persulcatus* ticks [2, 79, 88] and single exceptions were found in the sympatric area of *I. ricinus* and *I. persulcatus* located in Estonia and Latvia [79, 89]. Moreover, viral determinants responsible for the association of European and Siberian subtypes of TBEV have recently been revealed [90]. However, our observation of new genetic variants of *B. garinii* only in *I. pavlovskyi* ticks does not allow us to conclude that these variants are associated with this tick species because data on the genetic diversity of *B. burgdorferi* (*s.l*.) in *I. pavlovskyi* ticks are limited, and further detailed examination is required.

## Conclusion

In summary, the first detailed study of the prevalence and genetic characteristics of a wide range of infectious agents in *I. pavlovskyi* ticks demonstrated that almost all previously detected pathogens in *I. persulcatus* ticks can be found in *I. pavlovskyi.* Only *B. valaisiana* and *R. sibirica* were not found in the tested *I. pavlovskyi* ticks. For the first time, *B. bavariensis*, *R. helvetica*, *R. heilongjiangensis*, *R. raoultii*, “*Ca.* R. tarasevichiae”, *A. phagocytophilum*, *E. muris*, “*Ca.* N. mikurensis” and *Bab. microti* were identified in *I. pavlovskyi* ticks. For TBEV, KEMV, *B. miyamotoi*, *A. phagocytophilum*, “*Ca*. N. mikurensis”, and *Bab. microti*, the prevalence and genetic variants were similar in both *I. pavlovskyi* and *I. persulcatus* ticks. However, the distribution of species belonging to the *B. burgdorferi* (*s.l*.) complex, *Rickettsia* genus and *E. muris* was different between tick species, and many new genetic variants of *B. garinii* and *Rickettsia* spp. were identified in *I. pavlovskyi* ticks. In total, 58% of *I. pavlovskyi* and 88% of *I. persulcatus* ticks were infected by at least one of the examined agents (Table 3). *Ixodes pavlovskyi* ticks were significantly less often infected by pathogens compared to *I. persulcatus* ticks, because of the low prevalence of “*Ca*. R. tarasevichiae” in this tick species. We can assume that the *I. pavlovskyi*/*I. persulcatus* sympatric areas might be characterized by a greater genetic diversity of infectious agents and pose a greater threat to public health compared to the *I. persulcatus* allopatric areas, but further investigations of natural *I. pavlovskyi*/*I. persulcatus* hybrids are required.
